# Hydrogel applications: a promising frontier in pneumonia therapy

**DOI:** 10.3389/fbioe.2025.1602259

**Published:** 2025-06-20

**Authors:** Junming Wang, Pengfei Wang, Kehan Liao, Daikun He

**Affiliations:** ^1^ Department of General Practice, Jinshan Hospital, Fudan University, Shanghai, China; ^2^ Center of Emergency and Critical Care Medicine, Jinshan Hospital, Fudan University, Shanghai, China; ^3^ Research Center for Chemical Injury, Emergency and Critical Medicine of Fudan University, Shanghai, China; ^4^ Key Laboratory of Chemical Injury, Emergency and Critical Medicine of Shanghai Municipal Health Commission, Shanghai, China

**Keywords:** pneumonia, hydrogels, drug delivery, biocompatibility, intelligent responsiveness

## Abstract

Pneumonia remains a significant global health challenge due to its high incidence, mortality rates, and the limitations of conventional therapies, such as antibiotic resistance and inefficient drug delivery. In recent years, hydrogels have emerged as a promising biomaterial platform for pneumonia treatment, offering exceptional biocompatibility, tunable physicochemical properties, and multifunctionality. This review comprehensively examines the recent advancements in hydrogel applications for pneumonia therapy. It focuses on their roles as drug delivery vehicles, anti-inflammatory agents, and facilitators of tissue repair and regeneration. Hydrogels enable targeted and sustained release of antibiotics, anti-inflammatory drugs, and bioactive molecules, enhancing local drug concentrations while minimizing systemic side effects. Their ability to mimic the extracellular matrix (ECM) supports lung tissue repair and regeneration, addressing the long-term complications of pneumonia, such as fibrosis. Additionally, hydrogels can be engineered to respond to specific physiological conditions, such as pH or enzyme activity, allowing for intelligent drug release profiles tailored to the pulmonary microenvironment. Despite these promising developments, challenges related to material safety, drug loading efficiency, and scalability of manufacturing processes must be addressed to facilitate clinical translation. This review highlights the therapeutic potential of hydrogels in pneumonia treatment and provides insights into future research directions, aiming to bridge the gap between laboratory innovations and clinical applications.

## 1 Introduction

Pneumonia, a prevalent respiratory infection, presents a complex etiology involving various pathogens, including bacteria, viruses, and fungi. Characterized by symptoms such as cough, fever, chest pain, dyspnea, and fatigue (Scott et al., 2008), this disease has emerged as a critical global health challenge, affecting approximately 300 million individuals annually (Li et al., 2021) and directly causing over three million deaths worldwide (Ching and Pedersen, 2025). The burden disproportionately impacts vulnerable populations: elderly patients (>75 years) face high hospitalization mortality rates (30.3%) (Hespanhol and Barbara, 2020), while neonates account for 750,000–1.2 million annual deaths, representing 10% of global child mortality (Nissen, 2007).

Conventional therapeutic approaches, primarily comprising antibiotic therapy, respiratory support, and symptomatic treatment (Prina et al., 2015), face three major limitations. First, antibiotic therapy is increasingly compromised by the rise of multidrug-resistant pathogens (Cilloniz et al., 2021). A multi-country analysis estimates that the average prevalence of multiple-resistant *Staphylococcus aureus* (MRSA) in Asian hospitals reached 67.4% (Laxminarayan et al., 2016). Second, oxygen therapy fails to address underlying tissue hypoxia in severe cases (Klitgaard et al., 2023), while mechanical ventilation carries risks of ventilator-associated pneumonia (VAP) and barotrauma (Walter, 2021). Third, current symptomatic treatments (e.g., antipyretics, bronchodilators) merely alleviate symptoms without modulating the excessive inflammatory response that drives lung injury (Meijvis et al., 2012). Additionally, these approaches share systemic shortcomings: (1) inefficient drug delivery to infected alveoli due to poor pulmonary penetration and mucus barriers (Bassetti et al., 2020); (2) failure to promote alveolar repair (Singh et al., 2023), leading to long-term fibrosis in 28%–53% of survivors (Tran et al., 2022); and (3) inadequate coverage for viral and fungal pathogens, which account for 80% of cases in American children but lack targeted therapies (Jain et al., 2015). These limitations underscore the urgent need for novel therapeutic strategies to enhance pneumonia treatment outcomes (Doroudian et al., 2019).

In recent years, hydrogels have garnered substantial attention in biomedical research as a promising biomaterial platform, owing to their exceptional biocompatibility, tunable physicochemical properties, and multifunctionality. Composed of hydrophilic polymers forming three-dimensional networks through physical or chemical crosslinking, hydrogels can absorb and retain substantial amounts of water while mimicking the native tissue microenvironment (Cao et al., 2021). These unique characteristics have demonstrated remarkable potential in diverse applications, including drug delivery, tissue engineering, and regenerative medicine. Particularly in pulmonary disease management, hydrogels have emerged as a promising therapeutic tool due to their excellent mucoadhesive properties, controllable biodegradability, and capacity for localized sustained drug release (Wan et al., 2023).

In the context of pneumonia treatment, hydrogel-based strategies have demonstrated significant potential through several mechanisms: Firstly, hydrogels serve as efficient drug delivery vehicles, enabling targeted transport of antibiotics, anti-inflammatory agents, or bioactive molecules to infected pulmonary regions, thereby enhancing local drug concentrations while minimizing systemic side effects (Oliva et al., 2017). Secondly, their inherent physicochemical properties, including pH-responsiveness and enzyme-sensitivity, facilitate intelligent drug release profiles that can adapt to the complex pulmonary microenvironment (Ding et al., 2022; Hu et al., 2012). Furthermore, hydrogels can promote the repair and regeneration of damaged lung tissue by mimicking the structural and functional characteristics of the extracellular matrix (ECM) (Brown et al., 2022).

**FIGURE 1:**
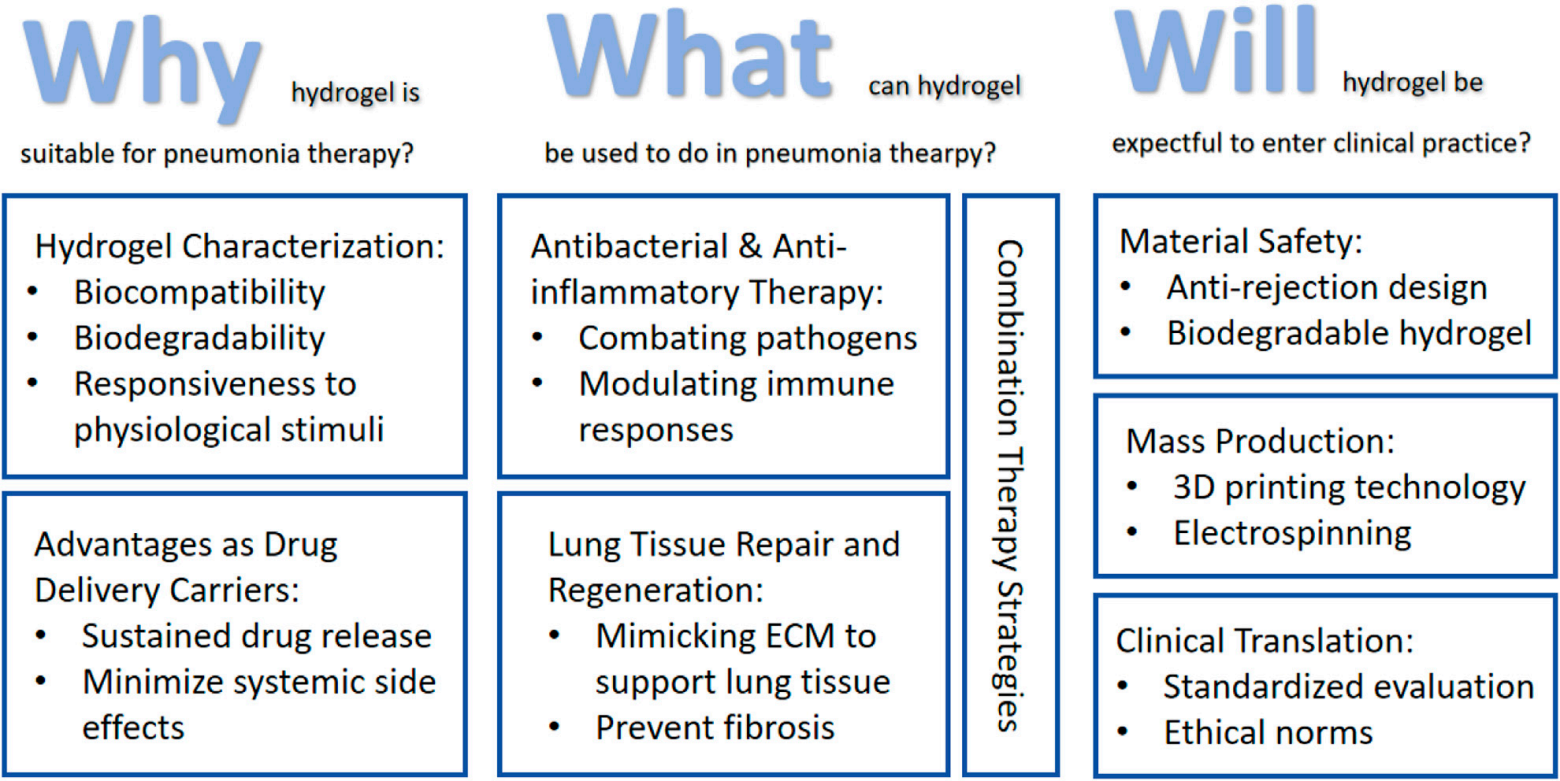
Roadmap of the main content discussed in this review: (1) Discuss the suitability of hydrogels for pneumonia treatment based on their intrinsic properties and advantages as drug delivery carriers; (2) Applications of hydrogels in pneumonia treatment encompass antimicrobial therapy, anti-inflammatory intervention, tissue regeneration, and comprehensive combination therapies; (3) Current limitations and challenges in hydrogel applications for pneumonia treatment, along with potential future improvement strategies.

Despite these promising advances in hydrogel-based pneumonia therapeutics, several challenges must be addressed before clinical translation, including material safety concerns (Souto et al., 2024), drug loading efficiency optimization (Chen et al., 2024), and scalability of manufacturing processes (Bernal et al., 2019). This review aims to comprehensively examine recent progress in hydrogel applications for pneumonia treatment, analyzing their mechanisms of action, therapeutic advantages, and future development directions, thereby providing both theoretical foundations and practical guidance for further research in this field (Figure 1).

## 2 Characterization of hydrogels and their suitability for pneumonia treatment

Hydrogels represent a unique class of crosslinked hydrophilic polymer networks with intrinsic porous structures that exhibit exceptional water retention capacity and closely mimic the natural extracellular matrix microenvironment (Zhu et al., 2023). Their distinctive architecture, combining high water content (often >90%) with tunable mechanical properties and biocompatibility, has driven remarkable progress in biomedical applications since their first clinical use in 1960s contact lenses (PHEMA hydrogel) (Mester, 1979). Over decades of development, hydrogels have evolved from passive biomaterials (Guowei et al., 2007) to sophisticated systems capable of stimuli-responsive behavior (e.g., pH-, temperature-, or enzyme-triggered changes) (Tian and Liu, 2023), enabling transformative applications ranging from targeted drug delivery (Mikhail et al., 2023) to advanced tissue engineering (Rastogi and Kandasubramanian, 2019) and bioelectronic interfaces (Yuk et al., 2019). The continuous innovation in hydrogel technology - from early wound dressings (Yang et al., 2021) to current self-healing formulations (Bertsch et al., 2023) and organ-on-a-chip platforms (Gnatowski et al., 2025) - demonstrates their unparalleled versatility as biomimetic materials that bridge materials science and cutting-edge medical therapies.

**FIGURE 2:**
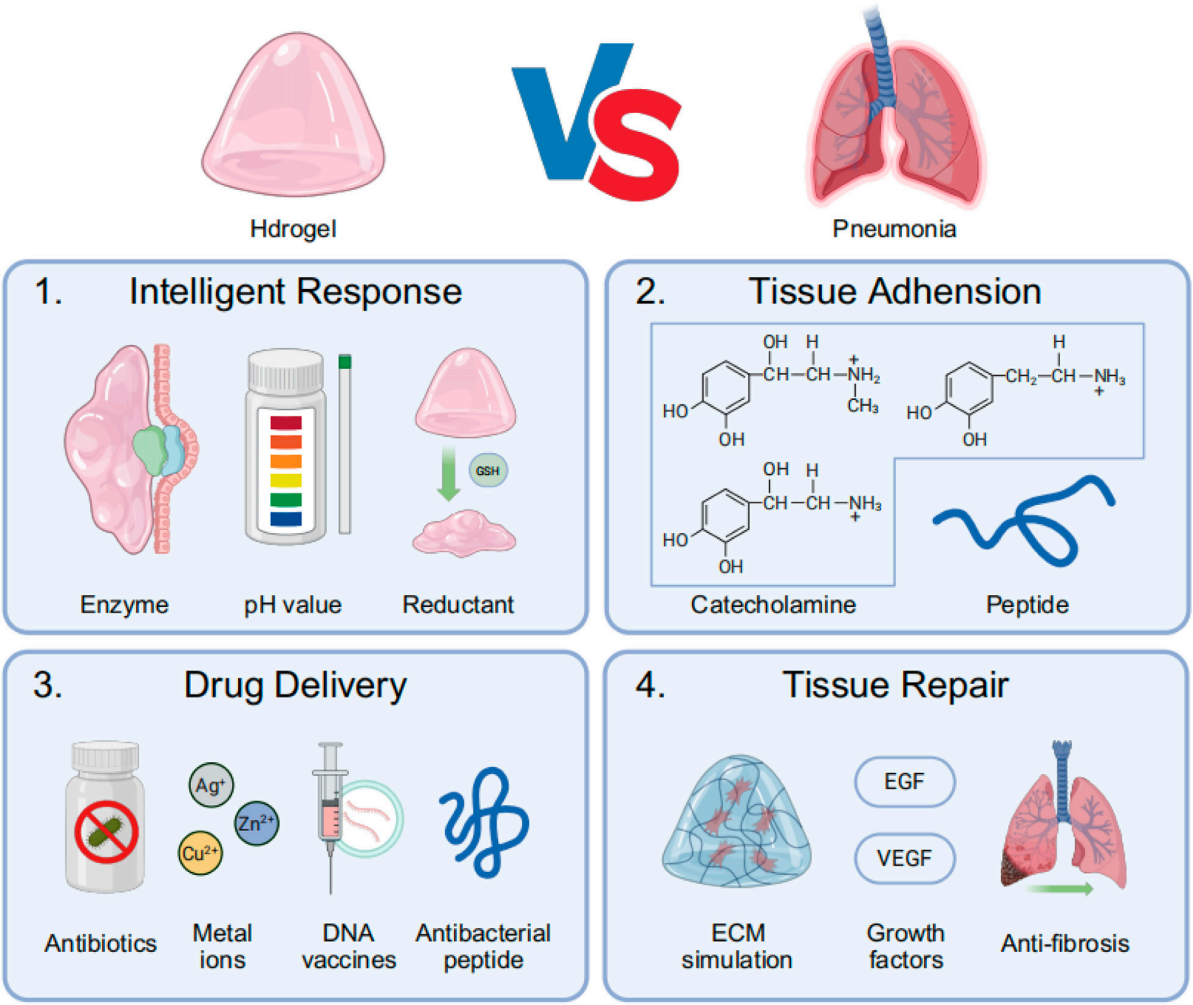
The main process of treating pneumonia with hydrogel: (1) Intelligent response: Hydrogels respond to infection-related local specific enzymes, low pH environments, or specific reductants such as glutathione (GSH); (2) Hydrogels achieve tissue adhesion through the conjugation of catecholamines and lung-targeting peptide motifs; (3) Hydrogels exert therapeutic effects by delivering antimicrobial agents, including antibiotics, metal ions, DNA vaccines, and antimicrobial peptides (AMPs); (4) Hydrogels promote post-infection lung tissue repair by mimicking the ECM while releasing growth factors and anti-fibrotic drugs.

Pneumonia’s unique pathophysiology—including viscous mucus obstruction, acidic infection sites, alveolar barrier damage, biofilm resistance, and dysregulated inflammation (Bohn et al., 2020; Lim and Siow, 2018)—makes hydrogel-based therapies particularly advantageous. Their high water content hydrates and loosens mucus (Hong et al., 2022), while pH-responsive swelling enables targeted antibiotic release in infected areas (Zhang et al., 2019). Hydrogels also mimic the lung’s ECM to support tissue repair, sustain drug delivery to overcome biofilm resistance, and modulate excessive immune responses (van Os et al., 2023). By combining mucolytic, antimicrobial, and anti-inflammatory functions in a single biocompatible platform, hydrogels outperform conventional treatments by providing localized, prolonged, and pathology-responsive therapy tailored to pneumonia’s complex biological mechanisms (Xiao et al., 2021) (Figure 2).

### 2.1 Physicochemical properties of hydrogels

Hydrogels are typically fabricated from natural or synthetic polymers, such as hyaluronic acid, chitosan, and polyethylene glycol (PEG) (Goor et al., 2017). These materials exhibit excellent biocompatibility and can interact favorably with lung tissue, thereby minimizing immune rejection (Nasra et al., 2023). For instance, a nanostructured hydrogel self-assembled from ciprofloxacin and tripeptide significantly reduced drug cytotoxicity while effectively killing Gram-negative bacteria (Marchesan et al., 2013). Another bioadhesive hydrogel (SHIELD) developed for severe acute respiratory syndrome coronavirus 2 (SARS-CoV-2) treatment showed no histological or functional damage in both *in vitro* and *in vivo* experiments (Mei et al., 2023). Unfortunately, at present, the research of this hydrogel mostly involves non-human primates with a small sample size, and the future clinical transformation is still far away. Furthermore, the biodegradable nature of hydrogels allows them to be gradually absorbed by the body after completing drug delivery or tissue repair, eliminating the need for metabolic detoxification or secondary surgical removal. A notable example is MICP-1, a mucin-based synthetic hydrogel that can block herpes simplex virus-1 (HSV-1) airway infection and rapidly degrade under reduced GSH treatment due to its abundant disulfide bonds (Bej et al., 2024).

The high water content of hydrogels (typically exceeding 90%) endows them with softness and elasticity similar to biological tissues, effectively mimicking the mechanical environment of the lungs (Shamskhou et al., 2019). Compared to artificial elastic membranes (e.g., polydimethylsiloxane, PDMS) used in traditional chip-based lungs, the F127-DA hydrogel component demonstrates a composition and stiffness more akin to human alveolar extracellular matrix (Shen et al., 2023). Additionally, when bovine lung extract-derived pulmonary surfactant was applied to a Carbopol hydrogel surface, it exhibited viscoelastic properties resembling natural, healthy tracheobronchial mucus (Schenck and Fiegel, 2016). These characteristics not only reduce mechanical damage to lung tissue but also provide a foundation for uniform distribution and adhesion in the lungs. However, the authors also admitted that they were still unable to fully control the factors affecting the pulmonary surfactant tension caused by Carbopol hydrogel.

The mechanical properties (e.g., elastic modulus, viscoelasticity), degradation rate, and drug release behavior of hydrogels can be precisely controlled by adjusting crosslinking density, polymer types, and functional modifications. For example, the FK-MEM@CMCO-CS hydrogel coating demonstrated optimal pH 5.0-dependent release of its conjugated AMP FK13-a1 and effectively controlled inflammatory factor production in a VAP rat model (Zhu B. et al., 2024). Notably, since a ventilator is a device requiring continuous use, this study did not address the long-term effects of the coating on host immune responses and the microbiome. Nadia A Mohamed et al. synthesized four novel crosslinked chitosan hydrogels (H1-H4) that showed significantly lower minimum inhibitory concentrations (MICs) against various common pneumonia pathogens compared to chitosan, all exhibiting pH and temperature responsiveness (Mohamed et al., 2019). Kasula Nagaraja et al. developed a pH-responsive TMGA-Ag nanocomposite hydrogel that effectively inactivated multidrug-resistant (MDR) bacteria, including both Gram-positive and Gram-negative strains (Nagaraja et al., 2021a).

Hydrogels can achieve specific adhesion to lung tissue through surface modification or functional design. For instance, hydrogels conjugated with polyphenol-based adhesion-triggering molecules such as catechol or pyrogallol groups can form strong bonds with pulmonary mucosa, thereby prolonging drug retention at infection sites. Nathan Gasek et al. developed two dopamine-conjugated hydrogels (ALG-MA-DA and GEL-MA-DA) that demonstrated excellent viscoelastic properties in *ex vivo* and *in vivo* preclinical pleural and tracheal injury models (Gasek et al., 2021). The authors claimed they were unsure why dopamine binding affects tensile strength and called for further mechanistic studies in the future. Another catechol-conjugated alginate (C-ALG) hydrogel localized around lung tissue within 5 min of *in vivo* injection and showed good cytocompatibility (Ji et al., 2021). Researchers also developed a tannic acid adhesion-triggering solution that, when combined with synthetic polyacrylamide-alginate (PAM/Alg) hydrogel, enhanced adhesion to porcine lung tissue by 45-fold (Li et al., 2024). Moreover, hydrogels can achieve targeted drug delivery by loading targeting molecules (e.g., antibodies, peptides). C16-ceramide, a unique receptor in pulmonary vascular endothelium, can be specifically recognized by hydrogels conjugated with endothelial lung-homing peptide (CGSPGWVRC) (Staquicini et al., 2023). Although the receptor protein of pulmonary vascular endothelium had individual heterogeneity, this study provided a new idea for targeting hydrogels. The advent of functionalized DNA hydrogels represents a groundbreaking development, as they can specifically recognize SARS-CoV-2 RNA signals in infected lung epithelial cells through transfer RNA (tRNA) triggered cascade signal amplification, releasing encapsulated particles that convert and amplify signals into colorimetric and temperature readouts for viral detection (Jiao et al., 2025).

### 2.2 Advantages of hydrogels as pulmonary drug delivery carriers

Hydrogels possess the unique capability to encapsulate drugs within their three-dimensional networks and facilitate controlled drug release through diffusion, swelling, or degradation mechanisms. This characteristic not only enhances local drug concentration in the lungs but also minimizes systemic exposure, thereby reducing side effects. For instance, antibiotic-loaded hydrogels can be delivered to the lungs via nebulization or direct injection, enabling sustained treatment or even prophylactic protection of infected areas. Khojasteh Shirkhani et al. developed a polymethacrylic acid (PMA) based anionic hydrogel capable of delivering amphotericin B (AmB) for effective treatment of aspergillus pneumonia (Shirkhani et al., 2015). The hydrogel nebulization route achieved a daily dosage of 135 μg/kg, demonstrating higher 8-day survival rates (100% vs. 80%) with only 75% of the conventional nebulization dose, proving to be a simple and cost-effective pre-exposure prophylaxis method (Schmitt et al., 1988). An agarose saline spray supplemented with 1% hydrogel significantly enhanced drug delivery to the posterior nasal region and effectively blocked SARS-CoV-2 binding to alveolar epithelial type II cells (AECII), providing short-term protection (Seifelnasr et al., 2024). Although the anthelmintic drug niclosamide shows therapeutic potential against respiratory viral infections such as SARS-CoV-2, respiratory syncytial virus, and influenza, its systemic side effects have limited its use. However, localized aerosol delivery through small PEG coating has enabled its deposition on airway epithelium (Ousingsawat et al., 2022). Furthermore, an injectable DNA hydrogel (NT-CpG) loaded with nano-toxoid was shown to promote Th1/Th17-biased immune responses, protecting mice from MRSA pneumonia and addressing antibiotic resistance (Guo et al., 2023).

The SARS-CoV-2 pandemic has significantly accelerated the development of hydrogel-based vaccines (Table 1). Several injectable hydrogel vaccines incorporating the receptor-binding domain (RBD) of the SARS-CoV-2 spike protein have been developed, including a polymer-nanoparticle (PNP) hydrogel system encapsulating RBD (Ou BS et al.) (Ou et al., 2023) and a hydrogel scaffold for prolonged poly (I: C)-adjuvanted RBD delivery (Chen J et al.) (Chen et al., 2022). A single-dose administration of these two systems in murine models induced sustained elevation of specific IgG titers for up to 28 days and enhanced gene expression lasting 185 days, respectively, demonstrating their capacity to elicit durable and effective humoral immune responses.

**TABLE 1:** Different types of hydrogel vaccines for presenting sars-cov-2 related antigens.

Type of hydrogel	Drugs carried	Administration method	Principle of controlled release	References
GelMA micro robot	Multiple DNA vaccines	Intramuscular injection	Magnetic drive	Chen et al. (2023)
Dynamic covalent hydrogel (DCH)	RBD protein antigen of SARS-CoV-2 original Wuhan strain and E2 protein of Nipah virus	Subcutaneous injection	The dynamic reversible covalent bond between the carrier and the loaded cargo is used to control the release	Shi et al. (2024)
p (APMA-THMA)hydrogel	SARS-CoV-2 RBD	Intravenous injection	Affinity of - NH_2_ and three - OH with RBD structure in hydrogel	Chen et al. (2022)
PEG-b-PLA hydrogel	SARS-CoV-2 RBD nanoparticles and RBD-16GS-I53-50	Subcutaneous injection	Retention of nanoparticles by hydrogel polymer networks	Ou et al. (2023)
Chitosan/GP hydrogel containing natural cowpea mosaic virus (CPMV) particles	Peptide sequence 809-826 of SARS-CoV-2 protein	Subcutaneous injection	CPMV coupled with epitope sequence 809-826	Nkanga et al. (2022)
Aphe-NP14 hydrogel	Wild type SARS-CoV-2 RBD	Intranasal spray	Aphe-NP14 has high affinity for wild-type RBD	Li et al. (2023)
PNP hydrogel	TLR9 adjuvant CpG, SARS-CoV-2 spike protein	Subcutaneous injection	CpG-NPs and SARS-CoV-2 spike proteins were immobilized on the hydrogel network with similar diffusion characteristics	Ou et al. (2024)
O_2_-Cryogel_VAX_ frozen hydrogel vaccine	SARS-CoV-2 nucleocapsid, RBD antigen, CpG adjuvant and GM-CSF	Subcutaneous injection	Polymer grids encase proteins and allow immune cells in and out	Colombani et al. (2021)

In addition to traditional single-drug hydrogel systems, hydrogels can also simultaneously load multiple drugs or bioactive molecules (such as antibiotics, anti-inflammatory drugs, growth factors, etc.), enabling synergistic therapy. As previously mentioned, FK-MEM@CMCO-CS can co-load meropenem (MEM) and FK13-a1 AMP, significantly reducing the required antibiotic dosage in the treatment of VAP (Zhu B. et al., 2024). Similarly, in tuberculosis treatment, an amphiphilic hydrogel drug delivery system, TB-Gel, can encapsulate a mixture of four first-line anti-tuberculosis drugs, achieving therapeutic efficacy equivalent to oral administration with only half the dosage (Pal et al., 2021).

The three-dimensional network structure of hydrogels can protect drugs from enzymatic degradation or pH variations, thereby enhancing drug stability. For instance, hydrogels loaded with protein or peptide drugs can prevent their rapid degradation in the pulmonary environment, prolonging their duration of action. For example, a hydrogel system synthesized by Christian Isalomboto Nkanga et al., coated with the SARS-CoV-2 protein peptide sequence 809-826, demonstrated stable release of 10%–12% over 21 days, in contrast to 100% release observed in phosphate buffer saline (PBS) (Nkanga et al., 2022). Additionally, Zhiyuan Shi et al. designed a dynamic covalent hydrogel (DCH) that achieved sustained release of loaded recombinant protein antigens for up to 30 days, while eliciting neutralizing antibody titers 150-fold higher than conventional Alum-adjuvanted vaccines (Shi et al., 2024).

Beyond serving as drug delivery vehicles, hydrogels can mimic the structure and function of the ECM, providing support for lung tissue repair. For example, hydrogels loaded with growth factors (such as VEGF, FGF) can promote angiogenesis and tissue regeneration, accelerating the repair of lung injuries. Chuang Hu et al. developed an epidermal growth factor (EGF)-loaded hydrogel (EGF@PP), which, when delivered as a dry powder via bronchoscopy to the injured site in a rabbit model, effectively accelerated airway epithelial repair (Hu et al., 2023). The bronchoscopy scores demonstrated 1.60-fold and 1.38-fold improvements over the control group on days 7 and 10, respectively. Another alginate/hyaluronic acid hydrogel loaded with basic fibroblast growth factor (bFGF), when injected directly into the larynx of aged rats, demonstrated a 1.5-fold increase in laryngeal muscle cross-sectional area after 12 weeks compared to controls (Choi et al., 2019). These findings suggest its potential for restoring swallowing function and preventing aspiration pneumonia in the elderly.

## 3 Advances in hydrogel applications for pneumonia treatment

Hydrogels, owing to their unique physicochemical properties and multifunctionality, have shown broad application prospects in pneumonia treatment. Below, we review recent research progress in this field from four aspects: antibacterial therapy, anti-inflammatory therapy, tissue repair and regeneration, and combination therapy strategies.

### 3.1 Antibacterial therapy

A key pathological feature of pneumonia is the colonization and proliferation of pathogens (such as bacteria, fungi, and viruses) in the lungs. As carriers for antibacterial drugs, hydrogels can significantly enhance local drug concentrations and prolong their duration of action, effectively inhibiting pathogen growth.

Hydrogels can load antibiotics (such as vancomycin, gentamicin, and ciprofloxacin) through physical encapsulation or chemical bonding, achieving sustained drug release via controlled mechanisms. For example, a hydrogel based on Methocel (hydroxypropyl methylcellulose) has been used to load amoxicillin, which, when administered orally to mice, significantly increased drug retention time and antibacterial efficacy at the infection site (Xu et al., 2017). Pradeep A et al. developed a chitosan hydrogel loaded with colistimethate sodium (a high-end antibiotic), which not only directly kills pathogenic bacteria but also effectively inhibits biofilm formation, reducing bacterial colonization potential (Pradeep et al., 2022). A novel hydrogel application strategy involves coating ventilator tubes with gentamicin-loaded hydrogel, greatly reducing the incidence of ventilator-associated pneumonia (Jones et al., 2015). Beyond traditional antibiotics, newly synthesized halogenated dopamine methacrylamides (DMA), particularly chlorinated DMA, when used in hydrogels, copolymers, and coatings, exhibit broad-spectrum antibacterial activity against multidrug-resistant bacteria, including MRSA, multidrug-resistant *Pseudomonas aeruginosa* (PAER), and carbapenem-resistant *Klebsiella pneumoniae* (CRKP) (Liu et al., 2021). Metal ions, due to their direct penetration of bacterial membranes, serve as excellent non-traditional “antibiotics.” Their ease of cross-linking with hydrogels has led to the development of numerous nanocomposites, such as Si@Ni (Gwon et al., 2024), Si@NiO-hydrogel (Gwon et al., 2022), ODHCs/CuONPs (Mohamed, 2024), and silver-coated hydrogels (Olson et al., 2002), which utilize metal ions as antibacterial components.

Antimicrobial peptides (AMPs) are small peptides with broad-spectrum antibacterial activity but suffer from poor stability and short half-lives *in vivo*. Hydrogels can protect AMPs from enzymatic degradation and enable their sustained release, enhancing therapeutic efficacy. For example, a polymyxin B (PMB) peptide-containing antibacterial hydrogel coating on ventilator tubes extended the release and antibacterial cycle to at least 42 days (Wouters et al., 2024). Similarly, applying a hydrogel containing neuranidase (CSA-131) to tracheal tubes has shown promising results in preventing VAP (Hashemi et al., 2018). A supramolecular nanogel structure formed by self-assembly of a poorly soluble antibiotic (ciprofloxacin) and a hydrophobic tripeptide ((D)Leu-Phe-Phe) demonstrated strong antibacterial activity against *Staphylococcus aureus* and *K. pneumoniae* in mice (Marchesan et al., 2013).

### 3.2 Anti-inflammatory therapy

Pneumonia is often accompanied by severe inflammatory responses, leading to lung tissue damage and dysfunction. Hydrogels can effectively mitigate inflammation and promote tissue repair by loading anti-inflammatory drugs or modulating the immune microenvironment.

Hydrogels can be loaded with glucocorticoids (e.g., dexamethasone), non-steroidal anti-inflammatory drugs (e.g., ibuprofen), or cytokine inhibitors (e.g., IL-6 inhibitors), achieving sustained drug release through controlled mechanisms. For instance, Zhen Wang et al. developed hyaluronic acid methacrylate hydrogel microspheres combined with genetically engineered membranes overexpressing angiotensin-converting enzyme II (ACEII) receptors. These microspheres compete with the SARS-CoV-2 virus for ACE2 binding, neutralizing pro-inflammatory cytokines, and calming the cytokine storm (Wang et al., 2022). In addition to their aforementioned antibacterial properties, the immunostimulatory effects of Ag + have also been extensively studied. Ag + can stimulate the production of a large number of leukocytes in the early stages of wound healing, generating synergistic antibacterial activity, followed by a rapid reduction in leukocyte count. This results in a shorter duration of inflammation compared to the normal process, minimizing tissue damage caused by inflammation (Zhang et al., 2023; Liang et al., 2024). Leveraging this property, a series of hydrogel materials embedded with silver nanoparticles have been developed, including TGIAVE-Ag (Nagaraja et al., 2021b), TMGA-Ag (Nagaraja et al., 2021a), and AgNPs-loaded CS/PVA hydrogels (Suflet et al., 2021). Furthermore, researchers have explored natural extracts for their anti-inflammatory potential. For example, hydrogels containing sea buckthorn extract not only exhibit strong antibacterial activity against *Streptococcus* pneumoniae but also possess anti-inflammatory properties (Nowak et al., 2022). Emulsified grape seed oil incorporated into hydrogels has shown inhibitory effects on Cyclooxygenase-1/2 (COX-1/2), with a predominant effect on COX-1 (Eid et al., 2025).

Hydrogels can exert anti-inflammatory effects by modulating macrophage polarization (e.g., promoting M2 macrophage activation) or inhibiting the release of inflammatory mediators. For example, a hydrogel carrying Y-27632, a Rho-associated protein kinase inhibitor, within CeO2-Y@ZIF-8@Gel, can repair damaged mitochondrial DNA, reduce the leakage of Ox-mtDNA, and downregulate the cGAS-STING pathway. This promotes macrophage polarization toward the M2 phenotype and enhances the production of anti-inflammatory factors (He et al., 2024). Another example is MNPs/Alg hydrogel, which inhibits oxidative stress damage by scavenging reactive oxygen species (ROS) and promotes macrophage polarization toward the M2 phenotype (Zhou et al., 2021). Although numerous hydrogels based on macrophage polarization modulation have been developed, their application in anti-inflammatory therapy for pneumonia remains underexplored. Therefore, this represents a promising direction for future research in the field of hydrogel-based pneumonia treatment.

### 3.3 Tissue repair

Lung tissue damage and fibrosis caused by pneumonia are among the most challenging aspects of treatment. Hydrogels, by mimicking the structure and function of the ECM, provide critical support for lung tissue repair.

The three-dimensional network structure of hydrogels can simulate the ECM of lung tissue, offering an optimal microenvironment for cell migration, proliferation, and differentiation. Current research has demonstrated that hydrogels derived from decellularized porcine lung ECM outperform traditional type I collagen and Matrigel in supporting cell adhesion, distribution, viability, and proliferation, making them a superior platform for three-dimensional cell culture and drug screening (Yan et al., 2022). Xinglong Zhu et al. developed a decellularized extracellular matrix (dECM) hydrogel, which was found to reverse pulmonary fibrosis in a mouse model by downregulating the ficolin signaling pathway and inhibiting M2 macrophage polarization (Zhu X. et al., 2024). Similarly, ECM hydrogels prepared from human lung fibroblast-derived matrix (hFDM) have shown significant potential for wound healing, although the underlying mechanisms remain incompletely understood (Savitri et al., 2020). Studies in rats have shown that ECM-derived hydrogels protect against lung injury through multiple mechanisms, including reducing epithelial-mesenchymal transition (EMT), inflammation, and oxidative damage (Zhou et al., 2020). Additionally, gelatin hydrogels that control the release of macrophage-regulating cytokines have successfully shifted macrophage polarization toward the M2 phenotype, promoting ECM structural and compositional changes and enhancing downstream remodeling potential (Witherel et al., 2021).

Hydrogels can be loaded with growth factors (e.g., VEGF, FGF, EGF) and promote angiogenesis and tissue regeneration through sustained release mechanisms. Functionalized dextran (FD), an anionic water-soluble polymer capable of binding transforming growth factor-β1 (TGF-β1), has been used to prepare dextran hydrogels that retain up to 88% of rhTGF-β1 (Maire et al., 2005). In a three-dimensional co-culture model of human umbilical vein endothelial cells and lung fibroblasts, a hybrid network of polycaprolactone-collagen (PCL/Col) and hyaluronic acid (HA) hydrogels loaded with VEGF supported not only cell attachment but also cell infiltration and the formation of primitive capillary networks within the scaffold structure (Ekaputra et al., 2011). Keiichi Hirose et al. synthesized micro-gelatin hydrogel microspheres containing bFGF, which, when administered intratracheally to rats, significantly increased pulmonary vascular density and improved hemodynamics (Hirose et al., 2008). Similarly, Qing Ban et al. modified an ordered colloidal crystal scaffold (CCS) made from chitosan and gelatin with bFGF, which greatly promoted the proliferation of lung epithelial cells and elicited only mild inflammatory responses when implanted into SD rats (Ban et al., 2019). Beyond direct growth factor delivery, encapsulating low-density lipoprotein receptor-related protein 5 (LRP5) in hydrogels has been shown to promote the expression of angiogenic factors and enhance compensatory lung growth in a mouse model of unilateral pneumonectomy (Mammoto et al., 2019). In addition to direct growth factor delivery, small interfering RNA (siRNA) inhalation therapy to activate endogenous regenerative programs is noteworthy. Hydrogels have effectively addressed the low intracellular delivery efficiency of siRNA. For example, hydrogel nanocomplexes combined with surfactant protein B (SP-B) have been reported as effective siRNA delivery enhancers, demonstrating efficacy in a mouse model of lipopolysaccharides (LPS)-induced lung injury (Merckx et al., 2018).

Hydrogels can inhibit pulmonary fibrosis by loading antifibrotic drugs (e.g., pirfenidone) or modulating the TGF-β signaling pathway. For instance, Elya A. Shamskhou et al. demonstrated that a hydrogel system based on hyaluronic acid and heparin, leveraging heparin’s reversible binding to Interleukin-10 (IL-10), could inhibit TGF-β-driven collagen production by lung fibroblasts and myofibroblasts (Shamskhou et al., 2019). Stem cell therapy holds significant promise for lung fibrosis regeneration but faces limitations, particularly the rapid clearance of implanted cells by the host. Hydrogel-based microcapsules for stem cell delivery have been shown to significantly prolong the persistence of donor mesenchymal stem cells (MSCs) in the host while enhancing their therapeutic functions, including immunomodulation and matrix metalloproteinase (MMP)-mediated ECM remodeling (Zhang et al., 2025).

### 3.4 Combination therapy strategies

The complex composition and multifunctionality of hydrogels enable the tailored construction of drug carriers based on the properties of the drugs. Through meticulous design and synthesis, hydrogels can easily integrate multiple drugs, combining antibacterial, anti-inflammatory, and tissue repair functions to achieve comprehensive pneumonia treatment.

The fundamental challenge of differing drug solubilities has been addressed, with novel hydrogels capable of accommodating both water-soluble and lipid-soluble drugs now reported. For example, a chitosan-crosslinked dialdehyde xanthan gum interpenetrating hydroxypropyl methylcellulose hydrogel has been developed for the controlled delivery of various antibiotics, including ampicillin, minocycline, and rifampicin (Ngwabebhoh et al., 2021). Researchers have also explored the use of adjuvants to enhance drug delivery. Paeoniflorin analog 1, a linear lipopeptide, significantly enhances the antibacterial activity of clarithromycin. Sun Hee Moon et al. co-encapsulated these agents in a hydrogel, achieving effective killing of multidrug-resistant *Acinetobacter* baumannii and *K. pneumoniae* (Moon et al., 2021). To comprehensively target different subtypes of the same pathogen, Yoshikazu Yuki and his team combined three pneumococcal surface protein A (PspA) fusion antigen monovalent formulations into a trivalent nanogel (Yuki et al., 2021). Additionally, the incorporation of toll-like receptors 9 (TLR9), which activate dendritic cells (DCs), and granulocyte-macrophage colony-stimulating factor (GM-CSF), which stimulates various immune cells, significantly increased antibody titers (Colombani et al., 2021).

Hydrogels can be combined with nanomaterials such as nanoparticles, liposomes, or exosomes to further enhance drug delivery efficiency and therapeutic efficacy. Beyond the traditional approach of integrating SARS-CoV-2 RBD domain nanoparticles with injectable polymer hydrogel systems (Ou et al., 2023), Ben S. Ou et al. designed a hydrogel-based SARS-CoV-2 vaccine by incorporating CpG nanoparticles (CpG-NPs), a potent TLR9 agonist, which greatly amplified the magnitude and duration of the antibody response (Ou et al., 2024). Another emerging area of research involves embedding vaccine silver nanocomposites into polymer matrices. Unlike the previously mentioned direct crosslinking of metal ions, Yi-Syuan Wei et al. developed a composite hydrogel containing only silver monomers, which not only exhibited excellent antibacterial properties but also demonstrated strong water absorption capabilities (Wei et al., 2016). Additionally, the combination of reduced graphene oxide (rGO) with hydrogels produced rGO@LNP, which exhibited both direct killing and adsorption effects against *K. pneumoniae* and *Enterococcus faecalis* (Sohni et al., 2023). In summary, the inherent versatility of hydrogels makes them a natural “adhesive,” offering researchers endless possibilities to harness the strengths of various materials.

## 4 Challenges and limitations

Despite the immense potential exhibited by hydrogels in the treatment of pneumonia, their practical application still faces numerous challenges and limitations. These issues encompass material safety, drug delivery efficiency, large-scale production, and clinical translation, among other aspects, all of which require further research and technological breakthroughs for resolution.

The biosafety of hydrogels in pulmonary drug delivery is a primary concern. Although most hydrogel materials, such as hyaluronic acid and chitosan, possess good biocompatibility, their long-term retention in the lungs may trigger local or systemic immune responses. For instance, certain synthetic polymeric materials, like polyacrylamide, may elicit inflammatory reactions or cytotoxicity (despite being useful in cancer treatment) (Bloch et al., 2017). Furthermore, prolonged retention of hydrogels in the lungs may affect lung function and even lead to fibrosis or other complications (Harding and Reynolds, 2014).

The physical properties of hydrogels, such as viscoelasticity and degradation rate, significantly influence their distribution in the lungs and drug release behavior. For example, higher viscoelasticity has been shown to reduce cell dispersion (Charrier et al., 2018) and may be difficult to uniformly distribute via nebulization inhalation; whereas hydrogels with too low viscosity may fail to effectively adhere to lung tissue (Lou et al., 2018). Furthermore, the degradation rate of hydrogels needs to match and remain constant with the drug release kinetics. Rapid degradation may lead to drug bolus release, while slow degradation may prevent the drug from reaching therapeutic concentrations. A notable example is the pulsatile release of parathyroid hormone, which stimulates bone formation, whereas continuous release leads to bone loss (Li et al., 2017).

The clinical translation of hydrogels faces numerous challenges, including the feasibility of large-scale production, drug loading efficiency and stability, and the realization of personalized treatment. The preparation process of hydrogels is complex and requires strict quality control standards, posing challenges for large-scale production. For instance, although microfluidic technology can produce uniform hydrogels, its production efficiency is low and difficult to meet clinical demands (Tien and Dance, 2021). Additionally, the drug loading efficiency of hydrogels may be affected by material properties, drug characteristics, and preparation processes, while drug stability within hydrogels (such as maintaining the activity of protein drugs) is also a critical issue (Lee et al., 2024). Finally, the etiology and pathological mechanisms of pneumonia are complex and diverse, and different patients may require different treatment regimens.

As a novel drug delivery system, the clinical translation of hydrogels requires rigorous regulatory approval (e.g., FDA, EMA). However, current regulatory standards for hydrogels are not yet comprehensive, adding uncertainty to their approval process (Wang et al., 2024). Currently approved medical tissue “adhesives”, including fibrin glue (Atrah, 1994) and cyanoacrylate (Trott, 1997), are not suitable for applications involving drug delivery. Furthermore, the long-term safety of hydrogels in pulmonary drug delivery has not been fully elucidated, which may raise ethical concerns. For example, in elderly patients or those with weakened immune function, the application of hydrogels may pose additional infection risks, requiring greater caution (Beattie et al., 2003; Lebaudy et al., 2021).

## 5 Future research priorities and potential innovations

To address the biosafety issues, researchers may attempt various approaches, including drug-eluting coatings (Farah et al., 2019), hydrophilic (Gudipati et al., 2005) or zwitterionic polymer coatings (Zhang et al., 2013), active surfaces (Dolan et al., 2019), and controlling material stiffness (Noskovicova et al., 2021) and/or size (Veiseh et al., 2015). Notably, Xuanhe Zhao and his team developed a hydrogel made of cross-linked polymers that effectively suppresses immune cell attacks on implants, a groundbreaking achievement published in Nature. Additionally, designing degradable hydrogels that can be naturally cleared by the body after completing drug delivery or tissue repair is also a crucial strategy to enhance their safety (Tian and Liu, 2023; Chen et al., 2023; Liu et al., 2023).

As for how to enhance the controlled release of hydrogel, researchers are optimizing the viscoelasticity of hydrogels by adjusting their cross-linking density or polymer concentration (Song et al., 2012; Wang and Windbergs, 2019) and achieving precise control over degradation rate and drug release through rational design of the hydrogel’s cross-linked network or introduction of stimuli-responsive groups (Numata et al., 2012; Hu et al., 2022). By optimizing the chemical structure of hydrogels, such as introducing protective groups to couple drugs (Nkanga et al., 2022) or adopting nanoparticle composite technology (Choi and Kohane, 2024), the drug loading efficiency and stability can be improved. These plans are all selective to drugs and lack simple, standardized synthesis methods. Although some novel materials supporting multimodal drug release have been reported (Day et al., 2022), there are still requirements for the standardization and large-scale production process of hydrogels in the future. To address this, researchers are developing efficient, low-cost preparation processes such as 3D printing (Mandrycky et al., 2016) and electrospinning (Xie et al., 2021), and establishing standardized quality control systems.

Finally, the booming development of molecular medicine research has raised new demands for personalized treatment. The development of smart responsive hydrogels, such as pH-responsive (Liang et al., 2022) and enzyme-responsive (Shigemitsu et al., 2020) hydrogels, or the integration of precision medicine technologies like genetic testing (Mo et al., 2021), can enable the individualized design of hydrogels to meet clinical needs. For these innovative personalized medications, Strengthening communication with regulatory bodies and establishing standardized evaluation systems for hydrogels are crucial for accelerating their clinical translation (James et al., 2023). Strict adherence to ethical norms in clinical trials and thorough assessment of the potential risks and benefits of hydrogels are essential prerequisites for ensuring their safe application.

## Glossary

MRSA: Multiple-Resistant *Staphylococcus Aureus*
VAP: Ventilator-Associated Pneumonia
ECM: Extracellular Matrix
PEG: Polyethylene Glycol
SARS-CoV-2: Severe Acute Respiratory Syndrome Coronavirus two
HSV-1: Herpes Simplex Virus-1
GSH: Glutathione
PDMS: polydimethylsiloxane
MIC: Minimum Inhibitory Concentration
MDR: Multidrug-Resistant
C-ALG: Catechol-conjugated Alginate
PAM/Alg: Polyacrylamide-Alginate
tRNA: Transfer RNA
PMA: Polymethacrylic Acid
AmB: Amphotericin B
AECII: Alveolar Epithelial type II Cells
RBD: Receptor-Binding Domain
PNP: Polymer-Nanoparticle
MEM: Meropenem
PBS: Phosphate Buffer Saline
DCH: Dynamic Covalent Hydrogel
EGF: Epidermal Growth Factor
bFGF: basic Fibroblast Growth Factor
DMA: Dopamine Methacrylamides
PAER: *Pseudomonas Aeruginosa*
CRKP: Carbapenem-Resistant *Klebsiella Pneumoniae*
AMP: Antimicrobial Peptide
PMB: Polymyxin B
ACEII: Angiotensin-Converting Enzyme II
COX-1/2: Cyclooxygenase-1/2
ROS: Reactive Oxygen Species
hFDM: human lung Fibroblast-Derived Matrix
EMT: Epithelial-Mesenchymal Transition
FD: Functionalized Dextran
TGF-β1: Transforming Growth Factor-β1
PCL/Col: Polycaprolactone-Collagen
HA: Hyaluronic Acid
CCS: Colloidal Crystal Scaffold
LRP5: Lipoprotein Receptor-related Protein five
siRNA: small interfering RNA
SP-B: Surfactant Protein B
LPS: Lipopolysaccharides
IL-10: Interleukin-10
MSC: Mesenchymal Stem Cell
MMP: Matrix Metalloproteinase
PspA: Pneumococcal surface protein A
TLR9: Toll Like Receptors nine
DC: Dendritic Cells
GM-CSF: Granulocyte-Macrophage Colony-Stimulating Factor
rGO: reduced Graphene Oxide
